# Diseases of the Digestive Organs in Infancy and Childhood

**Published:** 1901-12

**Authors:** 


DISEASES OF THE DIGESTIVE ORGANS IN INFANCY AND CHILDHOOD. With chapters on the diet and general management of children, and massage in pediatrics. By LOUIS STARR, M. D., late clinical professor of diseases of children in the Hospital of the University of Pennsylvania: consulting pediatrist to the Maternity Hospital, Philadelphia, etc. Third edition, rewritten and enlarged. Illustrated. Philadelphia: P. Blakiston's Son & Co. 1091. [Price, $3.00 net.]

In the ten years that have elapsed since the second edition of this book appeared, the changes in regard to many matters relating to pediatrics have been so great as to render the older volume almost obsolete. The present edition is therefore entirely rewritten. The first 100 pages are devoted to the general management of the child,—information of a kind more often presented to nurses and mothers than to physicians, but most valuable to the young practitioner. Then follow chapters on scurvy, rickets, and the various diseases of the digestive organs. The author's wide clinical experience forms the basis upon which the book is constructed and gives it a real value to the physician and pediatrist.

The book is handsomely printed and bound. M. J. F.

